# Higher skin sympathetic nerve activity as a potential predictor of overactive bladder in females refractory to oral monotherapy

**DOI:** 10.1002/kjm2.12899

**Published:** 2024-10-09

**Authors:** Yu‐Chen Chen, Hao‐Wei Chen, Tien‐Chi Huang, Chien‐Hung Lee, Ting‐Yin Chu, Yung‐Shun Juan, Yu‐Peng Liu, Wei‐Chung Tsai, Wen‐Jeng Wu

**Affiliations:** ^1^ Graduate Institute of Clinical Medicine, College of Medicine Kaohsiung Medical University Kaohsiung Taiwan; ^2^ Department of Urology, Kaohsiung Medical University Hospital Kaohsiung Medical University Kaohsiung Taiwan; ^3^ Regenerative Medicine and Cell Therapy Research Center Kaohsiung Medical University Kaohsiung Taiwan; ^4^ Department of Urology Kaohsiung Municipal Ta‐Tung Hospital Kaohsiung Taiwan; ^5^ Division of Cardiology, Department of Internal Medicine Kaohsiung Medical University Hospital Kaohsiung Taiwan; ^6^ Department of Public Health, College of Health Science Kaohsiung Medical University Taiwan; ^7^ Research Center for Environmental Medicine Kaohsiung Medical University Kaohsiung Taiwan; ^8^ NPUST College of Professional Studies National Pingtung University of Science and Technology Pingtung Taiwan; ^9^ Master of Health Care Management, Department of Business Management National Sun Yat‐Sen University Kaohsiung Taiwan; ^10^ Planning Office Kaohsiung Municipal United Hospital Taiwan; ^11^ Drug Development and Value Creation Research Center Kaohsiung Medical University Kaohsiung Taiwan

**Keywords:** autonomic nervous system, biomarker, overactive bladder, treatment outcome, urinary incontinence

## Abstract

This study investigates predictors of unsatisfactory outcomes in female overactive bladder (OAB) patients treated with oral monotherapy by analyzing skin sympathetic nerve activity (SKNA) using a novel “neuECG” method. The study included 55 newly diagnosed female patients with idiopathic OAB, autonomic function was evaluated using neuECG before treatment initiation, and validated OAB questionnaires and urodynamic studies were administered. Initial monotherapy was administered for the first 4 weeks, with non‐responders defined as patients not achieving satisfactory symptom relief and requiring further treatment. Responders (*n* = 32) and non‐responders (*n* = 23) had no significant differences in baseline characteristics or urodynamic parameters; however, non‐responders exhibited significantly higher baseline average SKNA (aSKNA) (1.36 ± 0.49 vs. 0.97 ± 0.29 μV, *p* = 0.001), higher recovery aSKNA (1.28 ± 0.46 vs. 0.97 ± 0.35 μV, *p* = 0.007), and a lower stress/baseline ratio of aSKNA (1.05 ± 0.42 vs. 1.26 ± 0.26, *p* = 0.029). Baseline aSKNA had the highest predictive value for monotherapy refractoriness in OAB (AUROC = 0.759, *p* = 0.001), with an optimal cut‐off point of >1.032 μV. These findings suggest that elevated pre‐treatment aSKNA can predict resistance to oral monotherapy in OAB, warranting close monitoring and proactive treatment strategies for patients with high aSKNA.

## 1 INTRODUCTION

The prevalence rates of overactive bladder (OAB) in women range from 9% to 43%, significantly impacting quality of life.^1^, ^2^ The European Association of Urology (EAU) and American Urological Association (AUA) guidelines recommend oral antimuscarinic or mirabegron monotherapy as a second‐line pharmacologic option for OAB treatment following first‐line behavioral therapies.^1^, ^3^ Despite the efficacy of oral monotherapy, a considerable proportion of OAB patients do not achieve satisfactory symptom relief and subsequently require further combination therapy with mirabegron and antimuscarinics or progress to third‐line treatment in 5%–10% of cases.^1^, ^4^, ^5^, ^6^, ^7^

While the AUA guidelines suggest assessing treatment efficacy within 4–8 weeks of initiating pharmacotherapy for OAB,^1^ this recommendation is classified as Level IV evidence due to its basis in expert opinion.^8^ There is insufficient consensus on when to transition to combination therapy or third‐line treatments for patients who are unresponsive to medication. This raises the question of whether all patients should adhere to the “wait and see” approach for 4–8 weeks to assess medication efficacy, prompting consideration of whether a more proactive and individualized treatment approach is warranted for OAB patients who do not respond to medication. Identifying predictors for unsatisfactory outcomes of oral monotherapy therefore becomes essential to optimize personalized treatment strategies to enhance patient outcomes.

Currently, there is a lack of robust predictors for assessing the efficacy of mono‐therapy in treating OAB, conceptualized as an imbalance in inhibitory and excitatory neural pathways to the bladder and the urethra, or sensitivity of bladder muscle receptors.^3^, ^9^ Recently, a novel noninvasive method called “neuECG” has been developed to simultaneously record skin sympathetic nerve activity (SKNA) and heart rate variability (HRV), providing an estimation of sympathetic tone in humans.^10^ Our previous research revealed significantly elevated SKNA in OAB patients compared to controls, suggesting its potential as a diagnostic biomarker for OAB^11^; consequently, we hypothesized that increased SKNA might contribute to treatment resistance and offer insights into potential therapeutic targets for non‐responders. This study aimed to investigate the association between ANS function and the response of OAB patients to oral monotherapy using neuECG, with the objectives being (1) to investigate whether there were significant differences in SKNA or HRV levels between the groups of patients responding effectively to medication and those showing ineffectiveness; and (2) if differences did exist, providing clinically relevant cutoff values that could potentially serve as predictors of monotherapy refractoriness in patients with OAB.

## 2 METHODS

This prospective study included female patients with newly diagnosed idiopathic OAB who were eligible for OAB drug therapy. OAB was diagnosed by the definition by the International Continence Society^12^ with exclusion criterion including the presence of urinary tract infections, neurological disorders, and cardiovascular disease. The primary outcome was the correlation between SKNA and HRV levels and treatment response, evaluated using a validated OAB symptom improvement questionnaire, with a secondary outcome aimed at determining whether these autonomic parameters could predict treatment response. This study was approved by the Institutional Review Board of Kaohsiung Medical University Hospital (KMUHIRB‐E(II)‐20220265). All participants provided written informed consent.

### 2.1 Urological assessment of OAB and the treatment effectiveness of monotherapy

All patients were assessed through history taking, OAB symptom score questionnaires (OABSS),^13^ International Prostate Symptom Score (IPSS),^14^ Urinary Sensation Scale (USS),^15^ and urodynamic studies (UDS) before treatment. The UDS examination was conducted with the patients in a sitting position; and patients were carefully monitored for bladder sensations during the filling phase, including the first desire to void, normal desire, strong desire to void, and cystometric bladder capacity (CBC). The voiding detrusor pressure (Pdet), maximum flow rate (Qmax), and post‐void residual (PVR) volume were recorded. If involuntary detrusor contractions occurred during the filling phase and reached bladder capacity, phasic detrusor overactivity (DO) and terminal DO were diagnosed, respectively.

Initial monotherapy, namely solifenacin succinate^16^ or mirabegron, was administered to all patients for the first 4 weeks. This cohort's primary urologists were two experienced urologists, both focused on Functional Urology. The treatment protocol included an evaluation at the fourth week of medication, conducted by one of these two urologists. Clinical efficacy was considered successful if the global response assessment (GRA score) after initial monotherapy was ≥1. This included improvements in symptoms from urge urinary incontinence to urgency, from urgency to frequency without urgency, or from any OAB symptom to being symptom‐free. Patients who did not experience any improvement in OAB symptoms after 4 weeks of treatment were classified as having failed initial medication and were subsequently switched to combination therapy or third‐line treatment options. Non‐responders were defined as patients who did not achieve satisfactory symptom relief and subsequently required combination therapy or third‐line treatment after the 4‐week period. The choice of monotherapy medication depended on the shared decision‐making process between the urologist and the patient.

### 2.2 Assessments of ANS

To acquire SKNA and HRV data, all participants were examined in a supine position during the morning in a controlled research environment with regulated temperature and humidity conditions, utilizing the neuECG methodology as detailed in prior literature.^11^, ^17^ Subjects were instructed to breathe at a rate of 10 breaths per minute. The signals obtained via neuECG were filtered at frequencies of 500–1000 Hz to display SKNA and 1–150 Hz to display electrocardiography (ECG). NeuECG recordings were conducted at baseline, during stress induction, and throughout the recovery phase, each lasting 5 min, for a total of 15 min. Stress phase was induced by mental arithmetic, specifically the subtraction of serial 13's from 1000, while participants were interrupted and urged to improve their performance.^18^ The definition of a normal sympathetic response is that sympathetic activity during the stress phase is higher than during the baseline and recovery phases. Any response that does not follow this pattern is considered an altered sympathetic response. Customized software was employed to analyze the average SKNA (aSKNA) per digitized sample, while the ratio of stress to baseline SKNA was designated as the sympathetic reserve.^18^

HRV analysis was performed using MATLAB‐based HRV analysis software.^19^ Time‐domain HRV parameters, including the standard deviation of normal‐to‐normal beat intervals and root mean square of successive differences, were calculated during the three phases (baseline, stress, and recovery), with each phase lasting 5 min. Frequency‐domain analysis determined the low‐frequency (LF) and high‐frequency (HF) components, as well as the LF/HF ratio.

### 2.3 Sample size estimation and statistics

The OAB patients who exhibited response and non‐response to oral monotherapy received the same treatment protocol but were assumed to have different baseline levels of SKNA. To achieve 80% statistical power in detecting a difference in population means of 0.25 for the SKNA level between OAB patients with and without oral monotherapy response, with a two‐sided type I error of 5%, a standard deviation of 0.24 for SKNA level (estimated from our previous study^11^), and an approximate response to non‐response patient ratio of 1:1, a total sample size of 30 participants (15 in each group) was determined.

Group differences were assessed using the chi‐square test for categorical variables and analysis of variance for continuous variables. All tests were two‐sided, with statistical significance set at *p* ≤0.05. Statistical analyses were carried out using IBM SPSS Statistics, version 26.

## 3 RESULTS

All subjects (55 individuals) completed the full 4‐week course of medication, with none discontinuing treatment due to adverse effects. Among them, 32 (58.2%) individuals responded to monotherapy after 4 weeks, while 23 (41.8%) individuals did not respond to monotherapy. Among those who did not respond, all 23 patients required a combination of antimuscarinics and mirabegron; however, 5 five patients had an inadequate response to the combination therapy and subsequently required botulinum toxin A injection.

Specifically, of the 27 individuals who used antimuscarinics as initial monotherapy, 13 (44.8%) did not respond and required further treatment. Of the 26 individuals who used mirabegron as initial monotherapy, 10 (38.5%) did not respond and needed additional treatment. There was no significant difference in efficacy between solifenacin succinate and mirabegron as initial monotherapy for OAB patients (Table 1). Baseline characteristics, encompassing age, frequency of urge urinary incontinence, questionnaire scores (OABSS, USS, IPSS, and IPSS‐QOL), and urodynamic parameters (first, normal, strong desire, CBC, the percentage of phasic or terminal detrusor overactivity, Qmax, Pdet, PVR), demonstrated no significant differences between responders and non‐responders to the monotherapy group (Table 1).

**TABLE 1:** Clinical and urodynamic parameters in responders and non‐responders to monotherapy group.

Variables	Responder	Non‐responders to monotherapy	*p*‐value
	*N* = 32	*N* = 23	
Age (years)	48.72 ± 12.35	53.78 ± 13.44	0.154
Urge urinary incontinence, *n* (%)	13 (40.6)	15 (65.2)	0.120
Urodynamic parameters			
First desire, mL (Mean ± SD)	144.71 ± 59.76	133.76 ± 73.14	0.567
Normal desire, mL (Mean ± SD)	227.79 ± 79.90	210.62 ± 89.89	0.484
The volume at strong desire, mL (Mean ± SD)	342.61 ± 137.55	277.95 ± 109.67	0.078
Cystometric bladder capacity, ml (Mean ± SD)	373.36 ± 146.81	338.77 ± 100.90	0.351
Compliance, mL/cmH_2_O (Mean ± SD)	65.27 ± 45.26	69.16 ± 39.32	0.756
Phasic DO, *n* (%)	19 (59.4)	12 (52.2)	0.321
Terminal DO, *n* (%)	27 (84.4)	21 (91.3)	0.394
Qmax, mL/s (Mean ± SD)	17.77 ± 8.33	17.36 ± 9.30	0.871
Pdet, cmH_2_O (Mean ± SD)	24.07 ± 14.97	27.10 ± 15.65	0.500
PVR, mL (Mean ± SD)	31.17 ± 44.43	31.91 ± 51.47	0.958
Symptom scores by validated questionnaires^a^			
USS	2.62 ± 1.12	2.40 ± 1.10	0.840
OABSS	7.03 ± 2.10	7.70 ± 2.34	0.590
Storage IPSS	7.93 ± 2.62	8.45 ± 2.84	0.790
Voiding IPSS	4.55 ± 4.89	5.90 ± 4.64	0.656
IPSS‐total score	13.83 ± 5.54	13.45 ± 5.63	0.706
IPSS‐QOL	4.07 ± 1.33	5.00 ± 1.17	0.071
Initial monotherapy used			
Solifenacin succinate, *n*, %	16 (50%)	13 (56.5%)	0.640
Mirabegron, *n*, %	16 (50%)	10 (43.5%)	0.640

Abbreviations: DO, detrusor overactivity; IPSS, international prostate symptom score; OABSS, overactive bladder symptom score; Pdet, detrusor pressure; PVR, post‐void residual; Qmax, maximum flow rate; QOL, quality of life; USS, urinary sensation scale; UUI, urge urinary incontinence.

^a^ The scores are presented as mean ± standard deviation.

### 3.1 Differences in SKNA and HRV between groups

**FIGURE 1:**
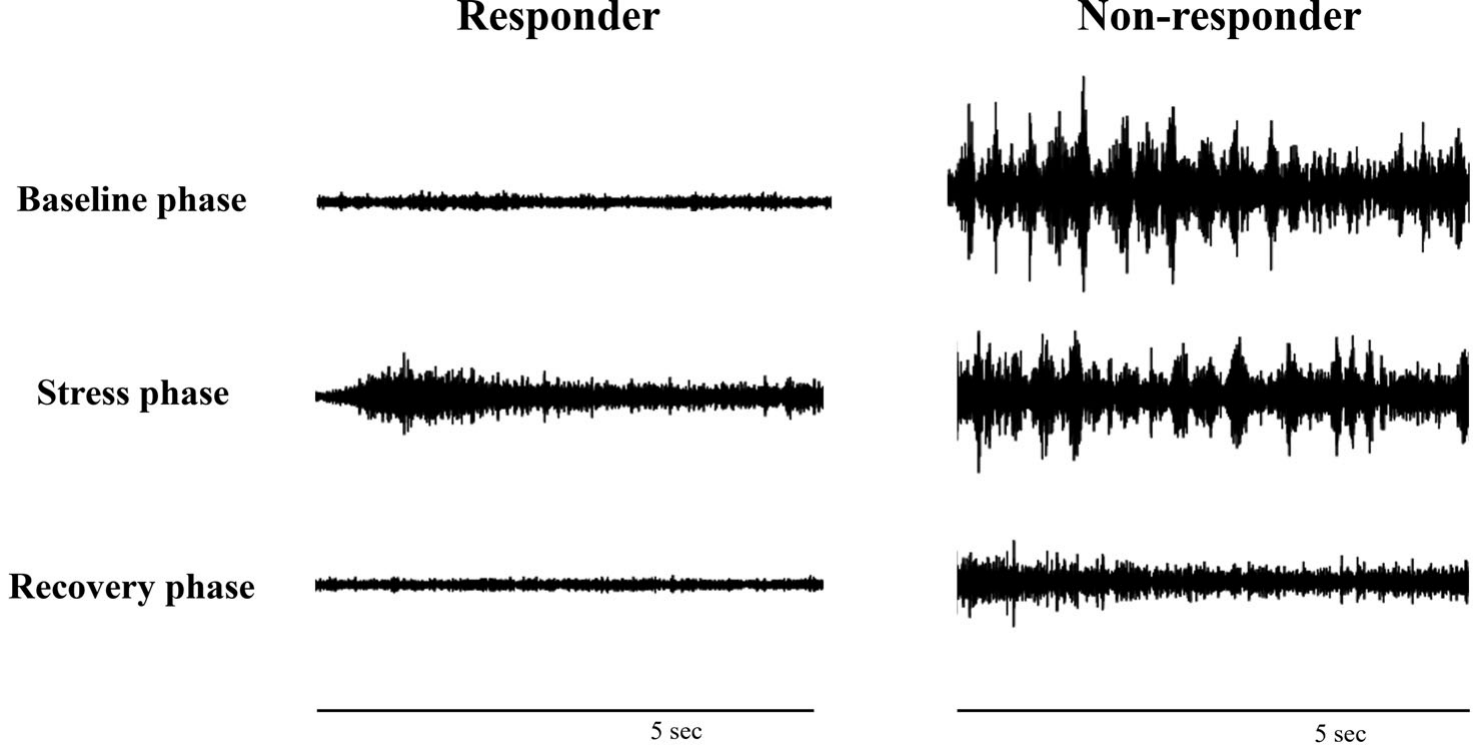
Original recordings of SKNA from a representative participant in responders and non‐responders. SKNA, skin sympathetic nerve activity.

Examples of SKNA recordings from responders and non‐responders groups are shown in Figure 1. The differences in aSKNA and HRV between these two groups in different phases are presented in Table 2. Non‐responders exhibited significantly higher baseline aSKNA (*p* = 0.001), higher recovery aSKNA (*p* = 0.007), and a lower stress/baseline ratio of aSKNA (*p* = 0.029); however, HRV‐related parameters did not differ significantly between groups. Table 3 compares the sympathetic response between responders and non‐responders to monotherapy. The non‐responders group exhibited a significantly higher rate of altered sympathetic response (*p* = 0.001).

**TABLE 2:** Comparison of autonomic function between the responders and non‐responders to monotherapy group.

Variables	Responder	Non‐responders to monotherapy	*p*‐value
	*N* = 32	*N* = 23	
	Mean ± SD	Mean ± SD	
SKNA			
Baseline aSKNA	0.97 ± 0.29	1.36 ± 0.49	0.001*
Stress aSKNA	1.18 ± 0.27	1.32 ± 0.44	0.154
Recovery aSKNA	0.97 ± 0.35	1.28 ± 0.46	0.007*
Stress/baseline aSKNA ratio	1.26 ± 0.26	1.05 ± 0.42	0.029*
Heart rate variability			
Baseline SDNN	37.61 ± 14.84	37.66 ± 19.84	0.992
Baseline RMSSD	30.24 ± 17.72	34.68 ± 26.69	0.462
Baseline LF	0.45 ± 0.16	0.40 ± 0.16	0.341
Baseline HF	0.55 ± 0.16	0.60 ± 0.16	0.341
Baseline LF/HF	0.98 ± 0.63	0.91 ± 1.00	0.744
Stress SDNN	49.84 ± 50.99	43.17 ± 26.07	0.568
Stress RMSSD	49.97 ± 72.93	43.48 ± 39.79	0.700
Stress LF	0.56 ± 0.15	0.55 ± 0.21	0.783
Stress HF	0.44 ± 0.15	0.45 ± 0.21	0.783
Stress LF/HF	1.64 ± 1.20	1.81 ± 1.66	0.660
Recovery SDNN	39.47 ± 16.31	39.42 ± 19.06	0.991
Recovery RMSSD	31.91 ± 17.76	32.52 ± 21.15	0.909
Recovery LF	0.44 ± 0.17	0.45 ± 0.17	0.806
Recovery HF	0.56 ± 0.17	0.55 ± 0.17	0.806
Recovery LF/HF	0.97 ± 0.70	1.01 ± 0.68	0.834

*Note*: *indicates a significant difference between the groups.

Abbreviations: aSKNA, average skin sympathetic nerve activity; HF, high‐frequency; LF, low‐frequency; RMSSD, the root mean square of the successive differences; SD, standard deviation;SKNA, skin sympathetic nerve activity; SDNN, the standard deviation of normal to normal beat intervals.

**TABLE 3:** Comparison of sympathetic response between responders and non‐responders to monotherapy.

	Responder	Non‐responders to monotherapy	*p*‐value
	*N* = 32	*N* = 23	
Normal sympathetic response, *n* (%)	26 (81.3%)	9 (39.1%)	0.001*
Altered sympathetic response, *n* (%)	6 (18.8%)	14 (60.9%)	0.001*

*Note*: The definition of a normal sympathetic response is that sympathetic activity during the stress phase is higher than during the baseline and recovery phases. Any response that does not follow this pattern is considered an altered sympathetic response.

* Indicates a significant difference between the groups.

### 3.2 Correlations between UDS parameters and SKNA

Table 4 highlights the correlations between skin sympathetic nerve activity (aSKNA) and various urodynamic parameters. Notably, we found significant correlations between the stress/baseline aSKNA ratio and the urge to void (first desire), the presence of phasic DO, and Pdet.

**TABLE 4:** Correlations between UDS parameters and skin sympathetic nerve activity.

Parameters	Baseline aSKNA	Stress aSKNA	Recovery aSKNA	Stress/baseline aSKNA ratio
First desire	n.s.	n.s.	n.s.	0.426
Normal desire	n.s.	n.s.	n.s.	n.s.
The volume at strong desire	n.s.	n.s.	n.s.	n.s.
Cystometric bladder capacity	n.s.	n.s.	n.s.	n.s.
Compliance	n.s.	n.s.	n.s.	n.s.
Phasic DO	n.s.	n.s.	n.s.	−0.609
Terminal DO	0.247	n.s.	n.s.	n.s.
Qmax	n.s.	n.s.	n.s.	n.s.
Pdet	n.s.	n.s.	n.s.	0.442
PVR	n.s.	−0.271	n.s.	n.s.

Abbreviations: aSKNA, average skin sympathetic nerve activity; DO, detrusor overactivity; Pdet, detrusor pressure; PVR, post‐void residual; Qmax, maximum flow rate.

### 3.3 Predictors of monotherapy refractoriness

**FIGURE 2:**
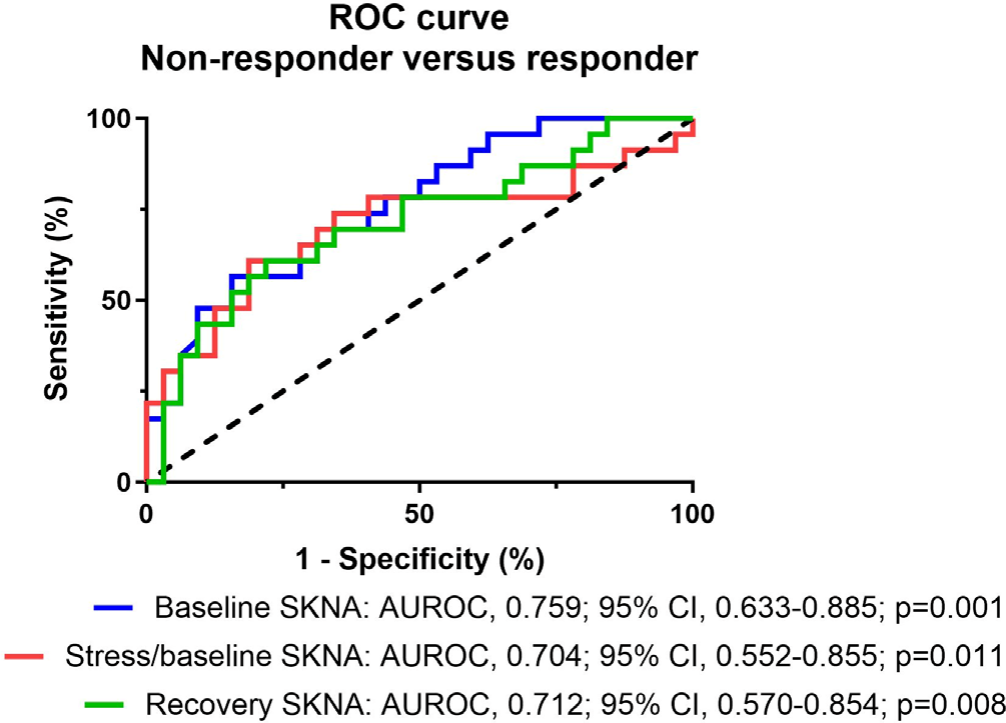
ROC curve analysis for predicting monotherapy resistance in overactive bladder (OAB). ROC curve analysis was conducted to predict the resistance of monotherapy in OAB using baseline phase SKNA, the ratio between stress and baseline SKNA, and recovery phase SKNA. Baseline SKNA exhibited the highest predictive value for monotherapy refractoriness. ROC, receiver operating characteristic; OAB, overactive bladder; SKNA, skin sympathetic nerve activity.

Figure 2 shows the ROC curve analysis using the different significant autonomic parameters to predict the resistance of monotherapy. Although both the recovery SKNA and baseline/stress aSKNA ratio could predict the monotherapy refractoriness (AUROC = 0.712 and 0.704, respectively), baseline aSKNA had the highest predictive value for monotherapy refractoriness in OAB (AUROC = 0.759, *p* = 0.001). A value of baseline aSKNA of >1.032 μV was the optimal cut‐off point for predicting monotherapy refractoriness in OAB (sensitivity, 70%; specificity 66%). The positive predictive value was 59.26%, while the negative predictive value was 75%.

## 4 DISCUSSION

Our study conducted a comprehensive assessment of autonomic function in the treatment of OAB, yielding novel findings. Specifically, we observed the following: (1) Non‐responders to oral monotherapy exhibited significantly elevated aSKNA, elevated recovery aSKNA, and reduced stress/baseline ratio of aSKNA; and (2) Baseline aSKNA emerged as the most predictive parameter for monotherapy refractoriness in OAB. These results suggest that aSKNA holds promise as a novel biomarker for evaluating the efficacy of OAB treatment while highlighting the limitations of HRV as a biomarker for assessing OAB treatment efficacy.

Our treatment algorithm adheres to guidelines set forth by the EAU and the AUA, initiating with oral monotherapy and advancing to combination therapy with antimuscarinics plus mirabegron for those who were refractory to monotherapy.^1^, ^3^ While the AUA guidelines suggest evaluating treatment efficacy within 4–8 weeks of initiating pharmacotherapy for OAB, our study protocol, in accordance with the BESIDE study and most clinical studies, opted to assess treatment response after just 4 weeks of single oral medication.^20^, ^21^ This evaluation aimed to discern whether patients should advance to combination therapy due to unresponsiveness or persist with single oral medication as a responsive group; nevertheless, consensus regarding the optimal assessment time for monotherapy efficacy and the optimal time for implementation of combination therapy or minimally invasive therapies both remain elusive.

Hence, the identification of predictors for treatment response to monotherapy in OAB is of paramount clinical significance. This approach facilitates tailored treatment for individual patients, circumventing unnecessary waiting periods for the efficacy of oral monotherapy and empowering physicians to proactively and confidently address OAB concerns. While established predictors such as age and disease severity provide valuable insights, our study delves into the autonomic dimension of OAB treatment response, uncovering a previously unexplored avenue.

While previous studies have explored the associations between the autonomic nervous system (ANS) and OAB using HRV,^9^, ^22^, ^23^, ^24^, ^25^, ^26^, ^27^, ^28^ suggesting a possible link between autonomic dysfunction and OAB, there is a notable absence of literature utilizing HRV to assess the efficacy of OAB treatments. Potential reasons for this include (1) the challenge of accurately assessing sympathetic activity, a key component of the ANS, via HRV due to the influence of both vagal and sympathetic activity on HRV measurements; (2) variations in the duration of HRV measurements across different OAB studies (ranging from 1 to 10 min), lacking a standardized measurement protocol^22^, ^23^, ^24^, ^25^; and (3) the limited temporal resolution of HRV, which operates on a minute‐by‐minute basis. NeuECG, capable of effectively measuring SKNA, overcomes the aforementioned limitations of HRV. Our prior utilization of neuECG in assessing OAB revealed significantly elevated SKNA levels in OAB patients compared to health controls, suggesting its potential as a diagnostic biomarker for OAB.^11^ Additionally, we found that SKNA levels decreased significantly post‐treatment.^11^ Building upon these findings, we further employed neuECG to investigate the relationship between SKNA and the efficacy of OAB treatments.

In the literature review, only one study had investigated the relationship between autonomic function and the effectiveness of OAB treatment utilizing sympathetic skin response (SSR): Ates et al. reported a significantly higher percentage of SSR absence in OAB patients refractory to oral anticholinergics, suggesting the potential utility of autonomic dysfunction in predicting antimuscarinic‐refractory patients,^24^ although the limitation of the SSR test lies in its binary nature, distinguishing only between presence and absence of response without providing quantitative measurements. Moreover, approximately 20% of patients demonstrating efficacy with drug therapy also exhibited SSR absence, indicating considerable limitations in its clinical applicability. Additionally, recent studies have explored the modulation of hypoxia and oxidative stress on bladder dysfunction, such as the work by Yilmaz‐Oral et al., which demonstrated the effects of co‐administering sodium hydrosulfide and tadalafil in a rat model of bladder outlet obstruction.^29^ This study highlights the potential for novel therapeutic approaches targeting oxidative stress and hypoxia in managing bladder dysfunction, which could possess implications for OAB treatment strategies.

Our current study methodology offers a practical approach applicable to patients preparing for pharmacological treatment initiation. By employing a pretreatment baseline aSKNA cutoff value of 1.032 (AUROC = 0.759), we can identify patients with baseline aSKNA levels exceeding this threshold for closer monitoring of treatment response. This approach might potentially obviate the need to wait for the 4‐week mark to consider combination therapy, thereby enhancing clinical management and patient outcomes. Nevertheless, further large‐scale studies and validation research are warranted for future refinement and validation of these findings.

Moreover, our study revealed that certain urodynamic parameters might correlate with sympathetic hyperactivity. The Stress/Baseline aSKNA ratio, referred to as “sympathetic reserve,” represents the extent to which SKNA can be activated during the stress phase.^30^ Sympathetic reserve tends to be high in two scenarios: first when baseline phase SKNA is low, and second when stress phase sympathetic activity is significantly higher than baseline activity. However, the majority of individuals typically fall into the first category. From the correlations in our cohort, we observe that sympathetic reserve is positively correlated with bladder volume to first desire. This suggests that when bladder volume to first desire is lower (indicating increased bladder sensitivity), sympathetic reserve is also lower (indicating higher baseline SKNA). Additionally, we found a significant negative correlation between the presence of phasic DO and sympathetic reserve (correlation coefficient = −0.609). This suggests that individuals with phasic DO have a lower sympathetic reserve, reflecting higher sympathetic activity during the baseline phase. These findings indicate a notable relationship between bladder sensitivity, DO, and sympathetic nerve hyperactivity.

Regarding the potential use of urodynamic parameters as precise predictive tools for OAB medication treatment, the current literature remains controversial. Although our cohort did not show significant differences in urodynamic parameters between responders and non‐responders, Wang et al. suggested that the presence of phasic DO may be associated with higher success rates of medications for treating OAB.^31^ Future research is needed to further explore this area.

In this study, we proposed analyzing SKNA as a potential biomarker for the treatment of OAB. While this innovative approach may provide valuable insights into the involvement of the sympathetic nervous system in OAB, it is important to consider the established research surrounding urinary biomarkers. Chancellor et al. have emphasized the significance of urinary biomarkers in the context of OAB and other lower urinary tract symptoms. Their study compared the concentrations of urinary inflammatory cytokines among patients with interstitial cystitis, OAB, urinary tract infection, and bladder cancer, highlighting the utility of urinary biomarkers in reflecting bladder pathology and guiding treatment decisions.^32^ Given the wealth of knowledge surrounding urinary biomarkers,^33^ future research should discuss how the analysis of SKNA as a biomarker aligns with existing studies. Furthermore, integrating findings from both SKNA and urinary biomarker studies could potentially lead to a more comprehensive understanding of the pathophysiology of OAB and enhance the personalization of treatment strategies.

Limitations of this study include a small sample size and the exclusive inclusion of female participants. The same treatment protocol was applied to all patients with OAB. The classification of patients into responders and non‐responders represents a comparison of subgroups derived from the same population receiving the same treatment, without specific baseline characteristics to clearly distinguish them. The sample size estimation was based on the assumption that the two subgroups of OAB patients had different baseline levels of SKNA, which were only measured pre‐treatment and not post‐treatment. Given the influence of gender on SKNA and the distinct etiologies of OAB between males and females,^30^ this article focuses solely on females; nevertheless, patients receiving solifenacin or mirabegron monotherapy were both included, as previous studies suggest comparable efficacy between these medications,^34^ and both the EAU and the AUA guidelines support these options as viable single‐agent treatment modalities.^1^, ^3^ Additionally, none of our participants discontinued medication within the four‐week period due to drug‐related adverse effects, although it is important to acknowledge that 4 weeks of therapy is a relatively short evaluation period. This is a significant limitation as it may not provide sufficient time for improvement, and should be considered when interpreting the results. Furthermore, the choice of monotherapy medication was dependent on the shared decision‐making process between the urologist and the patient, which introduces variability in the study. Different drugs can have varied effects on patients and SKNA, and this heterogeneity is a significant limitation. Future studies exploring biomarkers or prognostic factors should use a uniform intervention to minimize this variability and avoid compromising the results. We deliberately excluded neurogenic OAB from our cohort due to its distinct etiology from idiopathic OAB, allowing for focused investigation and discussion.

In summary, pre‐treatment aSKNA demonstrates a significant increase in idiopathic OAB patients resistant to oral monotherapy. These findings underscore the potential of aSKNA as a prognostic marker in OAB management. Elevated levels of aSKNA, particularly a baseline phase exceeding 1.032 μV, might serve as indicators of monotherapy resistance; accordingly, clinicians could utilize this information to tailor individualized treatment strategies, closely monitoring therapeutic responses and implementing proactive approaches. This personalized approach has the potential to enhance overall outcomes and quality of life for treatment‐resistant OAB patients. Understanding the role of SKNA in these individuals could inform more timely therapeutic decision‐making and improve clinical outcomes. Further research is needed to elucidate underlying mechanisms and validate the utility of aSKNA as a predictive biomarker in larger cohorts.

## CONFLICT OF INTEREST STATEMENT

All authors declare no conflict of interest.

## Data Availability

The datasets generated during and/or analyzed during the current study are available from the corresponding author on reasonable request.
